# Emerging role of complement in COVID-19 and other respiratory virus diseases

**DOI:** 10.1007/s00018-024-05157-8

**Published:** 2024-02-18

**Authors:** Mark T. Xiao, Calder R. Ellsworth, Xuebin Qin

**Affiliations:** 1 Division of Comparative Pathology, Tulane National Primate Research Center, Health Sciences Campus, 18703 Three Rivers Road, Covington, LA 70433 USA; 2 Department of Microbiology and Immunology, Tulane University School of Medicine, New Orleans, LA 70112 USA

**Keywords:** Complement, MAC, SARS-CoV-2, Respiratory syncytial virus, Influenza, Animal models

## Abstract

The complement system, a key component of innate immunity, provides the first line of defense against bacterial infection; however, the COVID-19 pandemic has revealed that it may also engender severe complications in the context of viral respiratory disease. Here, we review the mechanisms of complement activation and regulation and explore their roles in both protecting against infection and exacerbating disease. We discuss emerging evidence related to complement-targeted therapeutics in COVID-19 and compare the role of the complement in other respiratory viral diseases like influenza and respiratory syncytial virus. We review recent mechanistic studies and animal models that can be used for further investigation. Novel knockout studies are proposed to better understand the nuances of the activation of the complement system in respiratory viral diseases.

## Introduction

The complement system, a key component of innate immunity, provides the first line of defense against microbial invasion. It also participates in the pathogenesis of many chronic, non-infectious human disorders, including autoimmune diseases, hyperacute graft rejection, paroxysmal nocturnal hemoglobinuria (PNH), atypical hemolytic uremic syndrome (aHUS), and atherosclerosis [1, 2]. Since its first identification in 1895 [3], our understanding of the complement system has led to the development of successful complement-targeted therapeutics used to treat diseases ranging from PNH/aHUS to the SARS-CoV-2 infection (COVID-19) [4]. It is well established that bacterial invasion activates the complement system, forming activated bioproducts and the terminal membrane attack complex (MAC), a terminal complement activation product, thereby culminating in lysis and pathogen clearance [5]. However, it is less clear how viral infections such as SARS-CoV-2 activate the complement system. Like in SARS-CoV-2, the complement system appears to be maladaptive in respiratory syncytial virus (RSV) infection, which can similarly cause acute respiratory distress syndrome (ARDS), a life-threatening lung injury that allows fluid to leak into the lungs [6, 7]. However, in other respiratory viral infections that can cause ARDS, such as influenza A & B, the role of the complement system is complicated. C3 may be protective while C3a and C5a signaling and MAC may be detrimental to the host [8, 9]. The cellular and molecular mechanisms by which the components of the complement system contribute to the pathogenesis of these respiratory viral infections remain unclear. We will review (1) complement activation and regulation, (2) clinical evidence of the detrimental role of the complement in COVID-19, (3) beneficial effect of complement-related therapeutics on COVID-19, 4) the pathogenic roles of the complement in other respiratory viral infections including influenza and RSV, and 5) experimental approaches to dissect the complement-mediated mechanisms during acute respiratory viral infection.

### Complement activation and regulation

**Figure 1.**
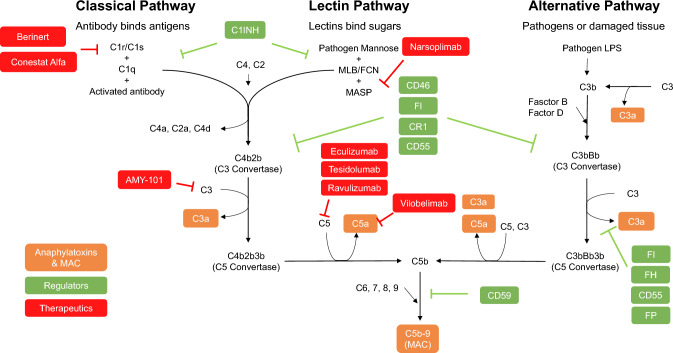
Complement (C) activation, regulation, and C-targeted therapeutics: Complement is activated by classical. Lectin and alternative pathways, which leads to forming the complement activation bi-products such as C3a, C5a, and membrane attack complex (MAC). Complement activation is restricted by an array of host complement regulators. The therapeutics listed include complement-targeted therapy used in clinical trials for the treatment of COVID-19. *FI* Factor I, *FH*, Factor H, *FP* Properdin, *C1INH, C1* esterase inhibitor, *LPS* lipopolysaccharide, *MLB* Mannose-Binding Lectin, *FCN* Ficolin, *MASP* Mannose-Associated Serine Protease. Adapted from: Lim et al. Blood Rev. (2023). Generated in Microsoft Powerpoint

The complement system is an essential component of the innate immune system. It has been evolutionarily preserved for hundreds of millions of years and comprises roughly 30 membrane-bound and soluble proteins (Fig. 1) [10, 11]. The complement system is activated by three distinct pathways that occur both on pathogenic surfaces and in plasma (Fig. 1) [1, 12]. The *classical* pathway is primarily triggered by antigen-bound antibodies. Specifically, and the Fc regions of these activated antibodies (primarily IgM and IgG) bind to C1q, which initiates the complement classical pathway [13–15]. Human IgG3 and IgG1 bind and activate C1 readily, whereas IgG2 does so poorly, and IgG4 exhibits no activity [14–16]. The *alternative* pathway can be stimulated by attachment of C3b, a cleavage product of complement component 3 (C3), to foreign particles and damaged tissue, or by spontaneous cleavage of C3. *The mannose-binding lectin (MBL)* pathway is initiated when the plasma MBL protein complexes with MBL-associated serine protease-1 and -2 (MASP-1/2), which in turn binds to microbial surface oligosaccharides and acetylated residues (Fig. 1) [17].

The three extracellular pathways (classical, alternative, and MBL) coalesce via the activation of C3 convertase and the cleavage of C3 to C3a and C3b [1, 12, 18]. This engenders a cascade of cleavage and activation events, including the cleavage of C5 to C5a and C5b via a C3b complex that culminates in the formation of the membrane attack complex (MAC; Fig. 1) [1, 12, 18]. MAC formation begins with the sequential recruitment of C6, C7, and C8 to C5b. The MAC embeds itself in the phospholipid bilayer, and C8 induces the polymerization of C9 molecules to form a pore-like structure. The MAC is a macromolecular pore capable of inserting itself into cell membranes and lysing foreign pathogens, and heterologous cells. Under certain pathological conditions such as PNH, the loss of complement regulators on erythrocytes leads to lytic MAC formation which can cause hemolysis [12, 19–21]. Additionally, the formation of MAC at sublytic concentrations in a cell membrane of nuclear cells such as monocytes and endothelial cells can stimulate signaling cascades [22–30] that lead to the activation of monocytes and mediate inflammation on blood vessel without the lysis of the cells [2, 31–34]. In addition to complement activation on cell surfaces and in serum, recent evidence points towards intracellular complement activation via local production, endocytosis, and phagocytosis [35, 36]. C3 and C5 cleavage has been observed in various immune cells, including lymphocytes, monocytes, and neutrophils, and may also have a signaling function [37].

To prevent excessive complement-mediated damage, several plasma and membrane-bound protein regulators have evolved to attenuate and restrict the complement system at different stages of activation (Fig. 1) [1, 12, 18]. Plasma factor I (fI) controls the production of active C3b by cleaving C3b into inactive iC3b and C3d [1, 12, 18] (Fig. 1). Factor H, also soluble, regulates the alternative pathway by accelerating the decay of C3 convertase and is a cofactor for factor-I-mediated inactivation of C3b [38]. The soluble C1 inhibitor regulates the classical pathway upstream [1, 12, 18] (Fig. 1). Membrane-bound protein regulators consist of complement receptor 1 (CR1), membrane cofactor protein (MCP) CD46, decay-accelerated factor (DAF) CD55, and CD59 (Fig. 1). These regulators are expressed on host cell membranes and protect the host from complement attack by inhibiting upstream complement convertases, deactivating complement products, and restricting the formation of MAC by inhibiting complement pathway activation at varying levels of the cascade [1, 12, 18]. For example, CD59 inhibits the formation of the MAC by directly binding to C8 and C9 and preventing the polymerization of C9, while CD55 accelerates the decay of C3 and C5 convertases [1, 11, 12, 18]. These regulators mediate a delicate balance between adaptive and toxic immune responses.

**Fig. 2:**
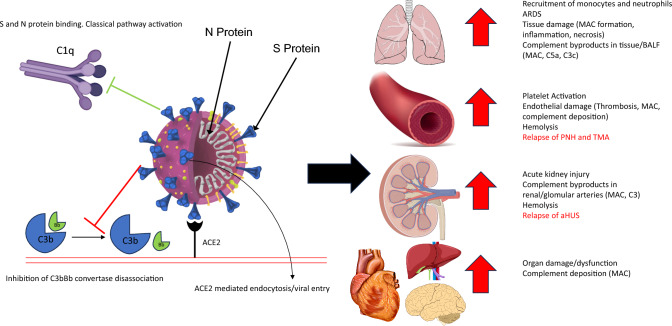
SARS-CoV-2 mediates complement activation and tissue damage and causes the relapse of complement dysregulation-related diseses: SARS-CoV-2 cellular invasion is mediated by ACE2 receptor binding. Beyond viral mediated cell lysis, SARS-CoV-2 can cause damage to tissues in the lungs, endothelia, kidneys, and other organs via the direct activation (and the inhibition of regulation) of the complement system. SARS-CoV-2 can also mediate the relapse of complement dysregulation-mediated diseases such as atypical hemolytic uremic syndrome (aHUS), Complement-mediated thrombotic microangiopathy (TMA) and paroxysmal Nocturnal Hemoglobinuria (PNH) (highlighted in red). *BALF* bronchiolar Lavage Fluid, *ARDS* Acute Respiratory Distress Syndrome. *S Protein* Spike Protein, *MAC* Membrane Attack Complex, *N Protein* Nucleocapsid Protein. Images used were imported from online sources under creative commons use licenses or generated in Microsoft Powerpoint.

Proper regulation of the complement cascade is essential for a healthy immune response. Over- or underactivation is associated with disease pathologies; for example, although PNH and aHUS are distinct in terms of clinical manifestation, they both feature a mechanistic overactivation of the complement system via various malfunctioning complement regulators including CD55, CD59, Factor H, and Factor I [39, 40]. Figure 2 details examples of SARS-CoV-2-mediates complement activation and tissue damage, using various images available online under creative commons use licenses [41–45]. On the other hand, some tumors may develop resistance to complement-dependent cytotoxic (CDC) chemotherapy via the overexpression of CD59 (and consequently, the inhibition of MAC) [46, 47]. CD59 also plays a large role in virulence: Increased incorporation of CD59 into viral envelopes can protect against antibody-dependent complement-mediated lysis in HIV-1, cytomegalovirus, and herpes, among other pathogenic viruses [48–53]. Respiratory viruses such as SARS-CoV-2, influenza, and RSV all show evidence of heightened activation of the complement system upon infection, but the consequences of this activation vary dramatically (as reviewed below). The mechanisms underlying these distinct host responses to specific infections are unclear and warrant further investigation.

### Increased complement activation is associated with severe COVID-19

COVID-19-related morbidity and mortality are increasingly thought to be related to excessive immune response [54]. While the pathogenesis of severe COVID-19 varies, key manifestations include platelet activation, thrombosis, endothelial dysfunction, immune activation, and cytokine storm [55–58]. Proteomic analysis from tissue samples obtained early in the pandemic identified complement overactivation as one of the strongest indicators of COVID-19 infection. For instance, hospitalized COVID-19 patients have significantly higher levels of circulating sC5b-9 than similarly sick influenza patients and C5a and alternative pathway markers are associated with increased COVID-19 severity [59]. In addition to the alternative pathway, which is predominant earlier in the course of diseases, clinical evidence points to complement activation across the MBL and classical pathways as well [60].

While evidence increasingly points to COVID-19 being a systemic disease [61, 62], pathogenesis of acute COVID-19 infection begins in and most severely impacts the lungs via respiratory failure and inflammation. Early clinical observations indicate heightened complement deposition in various tissue, including C3 and C5b-9 on lung endothelium and C5a in bronchoalveolar lavage fluid (BALF) [63, 64]. Heightened complement activity may also play a critical role in thromboinflammation in severe COVID-19 patients via the platelet/neutrophil extracellular traps (NETs)/thrombin axis [56] (Fig. 2). Coagulation and inflammation drive ARDS and contribute to the severity of the disease, and ARDS has long been associated with complement activation [65–67]. Some evidence suggests that complement activity may contribute to COVID-19 severity in a dose-dependent manner, with higher concentrations of sC5b-9 (solubilized MAC), C5a, and C3 byproducts in particular being associated with respiratory failure [57, 61, 63, 68–70] (Fig. 2).

The kidneys follow as the second most commonly affected organ system in COVID-19 patients, with an estimated 25% of patients hospitalized due to COVID-19 developing acute kidney injury [71, 72] (Fig. 2). Again, biopsies reveal that these patients had similar/elevated levels of complement activation products in renal and glomerular arteries relative to those with other complement-related kidney pathologies [73]. In fact, immunofluorescence staining of samples from nine patients with fatal COVID-19-related acute respiratory failure revealed that C5b-9 deposition patterns in the kidneys were remarkably similar to those in the lungs [64](Fig. 2). The similarity in COVID-19-related complement pathologies across organs suggests systemic complement overactivation. The complement system has been implicated in other COVID-19-mediated organ damage as well. For instance, deposits of various complement proteins have been observed in the livers, hearts, and systemic and cutaneous vasculature of infected patients [62, 64, 74–76] (Fig. 2). Together, extensive clinical evidence indicates that upon SARS-CoV2 infection, all three complement pathways can be activated and participate in the pathogenesis of severe COVID-19. SARS-CoV-2.

### COVID-19 induces the relapse of complement-related human diseases

As discussed previously, PNH and aHUS both feature a similar pathological under-regulation of the complement system via the altered expression or function of various complement cascade proteins (Fig. 2). Patients with preexisting irregularities are particularly sensitive to any level of complement overactivation. In one report, a 28-year-old female with previously diagnosed aHUS due to a heterozygous missense variant of the C3 gene suffered a relapse brought on by COVID-19 infection [77]. Other patients have been in similar situations, with COVID-19 and preexisting complement dysfunctions such as nonsense Factor H, CD46, and PIG-A mutations, and gain of function C3 mutations contributing to acute thrombotic microangiopathy (TMA) [78–81]. Utilization of eculizumab, a clinically used complement therapeutics to block C5a and MAC formation to suppressing complement activity in those patients showed immediate beneficent effects [77–81]. PNH and aHUS can be triggered by complement activation engendered by S protein antigens, and asymptomatic COVID-19 has also been reported to trigger multisystem inflammatory syndrome (MIS-C) in children (MIS-C), a rare, life-threatening complication of COVID-19 [82]. Decrease in complement activation is closely associated with rapid improvement of MIS-C after intravenous immunoglobulin treatment [82]. These cases further highlight the sensitivity of the complement system and how COVID-19 can disrupt the delicate balance between regulation and activation, thereby accelerating complement dysregulation-related diseases (Fig. 2). However, our understanding of the complement pathway activation on SARS-CoV-2 infection is limited and warrants further investigation. Therefore, in the following section, we will review the current understanding of complement pathway activation in COVID-19 and promising areas of investigation for future studies.

### Three pathways of complement activation in COVID-19

The general pathophysiology of acute severe COVID-19 consists of two successive phases: an initial viral invasion followed by an aberrant immunopathological response [7]. Viral invasion is facilitated by binding of the viral spike (S) protein to angiotensin-converting enzyme 2 (ACE2) cellular receptors [83] (Fig. 2). ACE2 is expressed in many tissue types, particularly in the lungs, heart, kidneys, brain, and endothelia [83]. This may account for the tropism in infection previously discussed, and the wide variety of corresponding symptoms that may occur. Tissue damage can stem from not only productive viral infection but also complement activation due to adjected infected tissue (particularly in the case of microvascular endothelial cells) [84].

### Classical pathway activation in COVID-19

The role of the classical pathway in severe COVID-19 is less investigated relative to the lectin and alternative pathways. While the classical pathway is associated with the adaptive immune response, it is notably not entirely antibody-dependent. For example, the classical pathway components C1q can bind directly to apoptotic blebs to initiate the complement cascade in bacterial infections [85, 86]. This seems to be consistent for SARS-CoV-2 as well, as anti-virus IgGs, S1, and N proteins bind to C1q and gC1qR [61, 70]. Additionally, complement activity assays using normal and C1q-depleted human serum have suggested viral protein and C1q binding in a dose-dependent manner [70]. Direct pathogen binding and complement reactive protein are both plausible factors for the activation of C1. Recent evidence has also highlighted a potential protective role of complement components C1q and C4b-bp. C1q produced by alveolar type II cells and local macrophages, independent of further complement activation, are known to modulate influenza A virus infection and replication, and ACE2 expressing cells infected with SARS-CoV-2 treated with C1q and C4b-bp were found to have reduced levels of inflammatory cytokines [87, 88]. This suggests that local, non-activating classical pathway activity may be beneficial to host defense. A deeper investigation using classical pathway protein knockouts is necessary to better understand how the classical pathway impacts the pathogenesis of severe COVID-19.

### Lectin pathway in COVID-19

The lectin pathway is clearly implicated in the pathogenesis of severe COVID-19. The concentration of lectin pathway-specific products, such as the MASP-1/C1-INH complex, have been correlated with COVID-19 severity [89]. Binding assays have shown that the SARS-CoV-2 N protein can interact with MASP-2, and complement deposition assays have linked N protein levels to activated C3 in a calcium-dependent manner [90] (Fig. 2). However, a separate cell study found that varying N protein treatment levels had no impact on cell viability, suggesting the possibility of sub-lytic complement activation via the lectin pathway [91]. MASP has also been implicated in animal studies of COVID-19. In one study, transgenic human ACE2-K18 mice susceptible to SARS-CoV-2 had improved symptomatic and phenotypic outcomes when treated with rescue HG4, an anti-MASP-2 antibody [92]. On the other hand, the role of the MBL protein is less clear. In a recent study from 2023, certain MBL polymorphisms (and lower functional MBL levels in general) were associated with a higher inflammatory response and disease severity [93]. This may be because MBL can suppress TNF-α and IFN-γ in natural killer cells and attenuate the acute immune response. Other MBL polymorphisms showed no correlation with any disease outcomes [89, 93]. Once again, the complement system’s activation-independent functions seem to play an adaptive role, as opposed to downstream complement activation effects. The net effects of complement components are not well understood; further investigation via single knockout animal models is warranted.

### Alternative pathway in COVID-19

The alternative pathway is continuously active at low levels and can amplify the response initiated by any pathway, providing a rapid response to pathogens. However, the rapid amplification of the complement cascade can be detrimental in certain disease contexts; the alternative pathway is disproportionately implicated in complement dysregulation-related diseases such as aHUS and PNH, among others [39, 94]. Evidence highlights that the alternative pathway is similarly implicated in COVID-19 complications; while complement products were generally upregulated among all patients, alternative pathway-specific biomarkers (such as increased byproducts including Ba/Bb and iC3b) and decreased alternative pathway regulator (properdin) in admission samples were correlated with increased severity and risk of mortality [60, 95]. Alternative pathway-specific SNPs, including factor B SNPs, have similarly been correlated with severity [96]. Clinical samples have highlighted a surge of factor D during the early phase severe COVID-19; in the human cell and macaque COVID-19 models, anti-factor D treatment mitigated complement activation and protected endothelial cells post-viral infection [97]. Enhanced C3 cleavage and downstream release of C3a and C5a may contribute to the severe COVID-19 phenotype by promoting myeloid cell infiltration and propagating inflammation.

These emerging clinical studies clearly indicate that SARS-CoV-2 infection activates three complement pathways. Interestingly, a recent study shows that SARS-CoV-2–encoded ORF8 protein may contribute to the decay of C3-convertase; this finding may help explain how the virus escapes elimination via the complement system and further highlights the gap between local and systemic complement responses [98]. SARS-CoV-2 may develop other mechanisms to inhibit the host complement pathway activation for escaping the complement attack, which requires further investigation. The relevance of local versus systemic complement response has been documented in bacterial pneumonia via a conditional complement knockout mouse model. Although the liver is primarily responsible for complement biosynthesis, complement production by lung epithelia relevant [99]. Using a tamoxifen-inducible Cre-Lox system, researchers selectively ablated the gene encoding C3 from lung epithelia and hepatic cells. Lung C3 knockout mice experienced greater acute lung injury caused by Pseudomonas aeruginosa infection than controls while maintaining near-normal levels of circulating C3. Conversely, mice deficient in liver-generated C3 had similar levels of the injury as wild-type controls [99]. Single-cell transcriptome analysis of the lungs of patients with COVID-19 has revealed that C3 is upregulated in various lung epithelial cells, including AT1, AT2, and club cells [100]. However, the distinction between local and systemic complement production and how they contribute to the hyperimmune activation seen in severe COVID-19 pathogenesis is unclear and also warrants further investigation. Regardless of the source, complement activation contributes to the overexpression of pro-inflammatory cytokines, including IL-6 and TNF-α, and worsening inflammation, organ damage, and general disease severity [56, 63, 101].

### Beneficial effects of complement therapeutics in COVID-19 patients

In this section, we will discuss the evidence for complement-related COVID-19 therapies, and how they may deepen our understanding of the pathogenesis of the disease. Specifically, these therapies highlight how introducing external complement therapeutics at different stages of the activation cascade can help attenuate the hyperinflammation engendered by COVID-19. For instance, downstream protein C5 has been a popular target due to preexisting complement therapies. In particular, the anti-C5 antibody eculizumab has shown positive effects in severe COVID-19 patients across various institutions (Fig. 1). Eculizumab, when used in conjunction with standard treatment in severe COVID-19 patients, improves survival rates and reduces comorbidities like hemolysis [102, 103]. While eculizumab may engender a drop in various inflammatory markers and reactive complement protein levels [77, 81, 103, 104], the associated drop in complement activity may in turn be associated with a greater incidence of infectious complications such as ventilator-associated pneumonia [103]. While eculizumab is currently the most extensively investigated complement inhibitor, other anti-C5 antibodies have also shown early success [105–107]. Tesidolumab, which binds to a separate C5 epitope, and ravulizumab, which shares the same epitope on C5 with eculizumab but has longer plasma stability, have also been investigated in preliminary single-arm studies of severe COVID-19 patients (Fig. 1).

Vilobelimab, an anti-C5a antibody, has also shown efficacy as a treatment for acute COVID-19 (Fig. 1). Vilobelimab, when used in conjunction with standard treatment, have shown improved survival outcomes [108]. Unlike eculizumab, vilobelimab can attenuate the anaphylatoxic and hyperinflammatory effects of complement activation without paralyzing terminal MAC formation nor upstream complement activation; this may be beneficial for patients with comorbidities or who are at risk for coinfection.

In contrast, upstream complement inhibition has also been investigated. Anti-C3 therapy via AMY-101, a compstatin analog, has been promising. Data from an FDA Phase 2 randomized controlled trial of severe COVID-19 patients indicated that AMY-101 was associated with reduced markers for inflammation and immunothrombosis without inducing further complications [109] (Fig. 1). Initial trials have shown that AMY-101 is comparable to eculizumab in reducing lung inflammation [110]. Berinert, a C1 esterase inhibitor (iC1e), has also been investigated as a potential therapeutic. iC1e is a human-derived protein used to treat hereditary angioedema primarily via the inhibition of the kinin-kallikrein system, but it also inhibits C1s and downstream classical pathway activity (Fig. 1). Conestat alfa, a recombinant rabbit-generated iC1e, has also undergone preliminary investigation. While preliminary investigation has produced mixed results, this approach can better isolate the effects of the classical pathway-specific response in severe COVID-19 [111, 112]. Narsoplimab, an anti-MASP-2 antibody, is a similarly upstream complement inhibitor that blocks lectin pathway activation. Narsoplimab treatment was associated with reduced circulating endothelial cell counts (a measure for endothelial damage), reactive complement proteins, inflammatory markers (IL-6, Il-8), and improved patient outcoomes [58]. All these trial studies further shed light on the important role of the complement pathway activation and complement activation bioproducts such as C3a, and C5a and MAC participate in the pathogenesis of severe COVID-19. However, the cellular and molecular mechanism underlying complement-accelerated severe COVID-19 remains unclear and requires further experimental investigation.

### Animal models for the investigation of the role of the complement system in coronaviruses

#### Animal models for the investigation of MERS-CoV and SARS-CoV

Complement activation not only occurs in COVID-19 patients but also in patients who are infected with Severe Acute Respiratory Distress Syndrome and Middle East Respiratory Syndrome (SARS-CoV and MERS-CoV), two similar viruses from the coronavirus family that can also result in acute lung injury and ARDS [90]. Increased complement activation has been suggested to play a critical role in the pathogeneses of severe SARS and MERS [113, 114]. Like SARS-CoV2’s N protein, the N proteins of SARS-CoV, MERS-CoV were also found to bind MASP-2, increasing complement activation by potentiating MBL-dependent MASP-2 activation, and the deposition of MASP-2, C4b, activated C3 and MAC [90]. While the three diseases are similar, SARS-CoV and MERS-CoV have distinctly higher reported fatality rates (9.5% and 34.4% respectively) [115, 116]. Additionally, MERS-CoV targets the DDP-4 receptor rather than ACE2 and is associated with a greater incidence of renal failure [117, 118].

Experimentally, SARS-CoV has been investigated using C3 knockout murine models. While there are no SARS-CoV-2 knockout studies published, investigators infected knockout and control (C56BL/6J) mice with a mouse-adapted SARS-CoV virus passaged 15 times [119]. They found that *C3*^−/−^ mice were most protected from SARS-CoV, while path-specific knockouts showed weak protection [119] (Table 1). This supports a multi-pronged approach toward COVID-related complement overactivation. *C3*^−/−^ mice did not display any weight loss following infection, and respiratory function was improved compared to controls. Staining and flow cytometry of lung tissue revealed much lower inflammatory monocyte and neutrophil populations [119]. Analysis of serum cytokines revealed that while infected control mice showed dramatic spikes in levels of IL-5, IL-6, CXCL1, and G-CSF on day two, their concentrations remained relatively constant over seven days of infection for *C3*^−/−^ mice, and there was no difference in viral load in the lungs of *C3*^−/−^ compared to control mice [119]. Further studies should clarify whether this phenomenon is present in COVID-19 as well and determine which complement-associated responses to SARS are essential for protection.

**Table 1:** Animal Models used

Virus	Complement associated harm or benefit	Author and Year	Complement factors investigated	Model details	Key findings
Coronaviruses					
SARS-CoV	Harmful	Lisa Gralinski (2018) [119]	C3	C3−/− and C57BL/6J control mice were infected with a mouse adapted SARS-CoV strain and compared against each other	C3 deficient mice had significantly reduced respiratory disease even though viral load was unchanged. Complement-deficient mice have reduced neutrophilia in their lungs and reduced systemic inflammation
MERS-CoV	Harmful	Yuting Jiang (2018) [120]	C5a/C5aR	Mice were genetically modified to express the hDDG4 receptor to facilitate MERS-CooV virus infection and replication.	MERS induced increased C5a and C5b-9 complement activation products in sera and lung tissues. anti-C5aR antibody treatment led to decreased viral replication in lung tissues, and increased proliferation in the spleen
SARS-CoV2	Harmful	Youssif M Ali (2018) [92]	Lectin pathway	K18-hACE2 transgenic mice	Administration of HG4, an anti-MASP-1 antibody significantly reduced the lung injury score including alveolar inflammatory cell infiltration, alveolar oedema and alveolar haemorrhage.
SARS-CoV2	Harmful	Eri Kawakami (2023) [97]	Alternative pathway	SARS-CoV2-infected rhesus macaques.	Anti-complement factor D monoclonal antibody mitigated abnormal complement activation, protected endothelial cells, and curtailed the innate immune response post-viral exposure
RSV					
	Harmful	Fernando Polack (2002) [159]	C3	C3−/−, B-cell deficient, and BALB/c control mice immunized with a formalin inactivated RSV and subsequently were challenged by RSV.	C3 deficient mice had lower airway hyperresponsiveness, but similar lung inflammation as their WT counterparts. The complement system plays an important role in the immune complex mediated damage from RSV
	Harmful	Monali Bera (2011) [6]	C3a/C3aR	C3aR1−/− mice were infected with a human RSV strain and compared against BALB/c control mice.	C3aR deficient RSV-infected mice had similar airway sensitivity as healthy controls. Bone marrow transplantation from C3aR −/− donors engendered a rescue phenotype in WT mice who were infected with RSV
	No effect or protective	Alexander Bukreyev (2012) [160]	C3, C5 (Cobra venom)	WT BALB/c mice were treated with either cobra venom factor to deplete complement levels, or a saline control.	The complement system does not reduce RSV viral titers in the lungs of RSV naïve animals. However, it plays a role in antibody-mediated complement cytotoxicity.
Influenza A					
	Beneficial	Manfred Kopf (2002) [8]	C3, Cr1, Cr2	C3−/−, Cr2−/−, and control C57BL/6 mice were infected with influenza (PR8) and then compared.	C3 plays an important role during influenza infection via the induction of T-cell lymphocyte priming and migration.
	Beneficial	Kevin O'Brien (2009) [164]	C3, C5a, iC3b	C3−/− and wt C57BL/6 mice were infected with influenza (PR8) and compared	C3 deficiency was associated with delayed viral clearance, lower morbidity, as measured by body weight, and lower evidence of histological damage. No elevated C3 levels in mice infected with PR8 or CA09
	Harmful	Shihui Sun (2013) [164]	C3aR, C5a and	H5N1-infected mice treated with C3aR antogonist, anti-C5a antibody, or cobra venom factor	treatment of H5N1-infected mice with a C3aR antagonist, anti-C5a antibody or with cobra venom factor significantly reduced acute lung injury
	Harmful	Nianping Song (2018) [162]	C5a-C5aR1, C3, MAC	C5aR1−/− and control BALB/c mice were infected with a lethal dose of influenza(PR8) and then compared.	C5aR1 deficiency was associated with less lung damage and inflammatory markers, although overall survival and body weight were unchanged
	Harmful	M. Paula Longhi (2007) [165]	MAC	mCd59a−/− mice were infected with influenza virus, strain E61-13-H17	CD59a, previously defined as a complement regulator, modulates both the innate and adaptive immune response to influenza virus by both complement-dependent and -independent mechanisms

The complement system’s role has also been investigated in mouse models of the MERS-CoV [120]. hDPP4 transgenic mice were infected with MERS-CoV and administered an anti-C5aR antibody or sham treatment (Table 1). Histopathological analysis revealed antibody treatment was associated with alleviated lung damage, though there were no differences in overall survival. In contrast to SARS-CoV, anti-complement treatment was associated with lower viral load in the lungs and brain, but it was unclear whether these inconsistencies were driven by differences in the pathogen or the mouse model used in the experiments [120]. Inhibition of the C5a-C5aR axis also reduced splenic damage (measured by caspase-3 and TUNEL staining) and increased T-cell regeneration (measured by PCNA) in red pulp (parenchymatous tissue of the spleen that consists of loose plates or cords infiltrated with red blood cells) [120]. Viral replication did not occur in the spleen (as hDPP4 expression, essential for viral uptake, was low), indicating that the damage induced by the complement system is at least partially systemic in nature. Although there are notable differences between MERS and SARS infection, overactivation of the complement seems to be a common factor. While these pathogens are distinct from SARS-CoV-2, understanding the similarities between the three will improve our understanding of the complement system and better equip us to deal with future coronavirus outbreaks.

#### Animal models for COVID-19

In addition to mice, Syrian golden hamsters and rhesus macaques also show promise as infection models for SARS-CoV-2. Like mice, these animals show upregulated markers of complement activation following acute COVID-19 infection [121, 122]. Golden hamsters and rhesus macaques typically recover from SARS-CoV-2, so they may serve as potential alternative animals for non-lethal models [123]. Hamsters and nonhuman primates share a structural similarity in their ACE2 receptor that closely resembles that of humans and can be directly infected with the human virus strain, which could more closely replicate human COVID-19 infection and inform the design of antiviral antibodies. Complement factor D antibody reduces endothelial dysfunction, cytokine, and coagulation in the primate COVID-19 model, which highlights the importance of alternative pathway activation in COVID-19 as discussed above [97] (Table 1). Nonhuman primate models most closely mimic human disease phenotypes, so they are particularly useful for developing new therapeutics and experimentally evaluating the mechanisms of COVID-19 and long COVID-19, however, unlike mice, few tools are available to manipulate hamsters. and rhesus macaques are costly and can be logistically difficult to manage. Mice remain the most suitable models for a sequential investigation of complement component knockouts and illuminating the nuances of complement function.

SARS-CoV-2 does not recognize the mouse ACE2 receptor, and as a result, mice are not susceptible to severe infection by ordinary means. Current mouse models utilize different strategies; for example, K18-hACE2 (K18) transgenic mice express the human ACE2 receptor under the keratin 18 promoter to induce expression in epithelia. This facilitates viral entry in the airway, simulating how infection begins in humans. SARS-CoV-2-infected K18 mice recapitulate various COVID-19 phenotypes seen in humans, ranging from moderate to severe disease and even death [124–126]. The meta-analysis of single-cell data across five model species showed that the K18-hACE2 mouse model, followed by the hamster model, most closely resembled human COVID-19 lung pathology [127]. The severity of the disease in this model is dependent on the infectious dose of the SARS-CoV-2 virus [128]. As discussed above, the inhibition of the lectin pathway of complement activation with anti-MASP-2 antibody (HD4) reduces ARDS severity in SRAS-CoV2-infected K18-hACE2 mice [92] (Table 1). This model has been widely used to evaluate the efficacy of potential therapeutics and vaccines; however, it is rarely used for mechanistic study, as the level and pattern of hACE2 expression in K18 mice differ from that of humans. While hACE2 expression in lung epithelia is similar in effect across both species, mouse models may not accurately reflect its level of expression across different organs in humans. For instance, hACE2 expression in mouse brain may be elevated compared to human brain tissue [129].

Other researchers have developed a non-lethal mouse-adapted (MA) strain via a serial passage in BALB/c mice lungs, selecting virulent strains. After 10 passages, one strain (MA10) was identified that showed high virulence in standard laboratory mice without decreased fitness in human cells [130]. Importantly, this model imitated the age-dependent severity we see in humans, with older BALB/c mice having more severe lung damage, weight loss, and mortality. While this strain had high pathogenicity in BALB/c mice, it was mild in C57BL/6 mice. The highest dose tested (10^5^ PFU) engendered a transient ~ 10% loss in weight, but no mortality.

In 2021, a more lethal mouse-adapted strain was identified after 30 passages (MA30) through 8–10-week-old BALB/c mice. Among other differences, the MA30 strain showed spike protein mutations, including Q498R, Q493R, and K417M, all of which are suspected to enhance mACE2 binding [131]. Mutations in the S protein receptor-binding domain may limit the viability of this approach for antibody development, as this region is a primary target for neutralizing antibodies. A 5*10^4^ PFU dose of the MA30 strain was highly lethal in young (6–10 weeks) BALB/c and middle-aged (6–9 months) C57BL/6 mice, and non-lethal in young C57BL/6 mice [131]. The range in severity makes this a suitable strain for C57BL/6 mice [131]. Viral load was present but attenuated in other organs relative to the lungs. In K18-hACE2 mice, viral load in many organs is comparable to that of the lungs, including the heart and brain [129]. This may be because mACE2 expression more closely replicates ACE2 expression in human tissue than the transgene inserted under the K18 promoter. The C57BL/6 mouse strain is widely used for investigating the pathogenesis of human disease in mice. C57BL/6 mice can be infected by the MA30 strain directly and have been crossed with a number of transgenic and knockout mice to introduce molecularly engineered genes in the C57BL/6 background. As such, MA30 can be easily used to dissect the pathogenesis of severe COVID-19 and potentially long COVID-19 as well.

#### Dissecting the role of complement and complement activation in COVID-19 using complement knockout mice

While the MA COVID-19 strains and hACE2-K18 mice are useful for pathogenic studies, substantial species differences in complement and complement-regulatory proteins exist between humans and mice and must be considered in the application of a mouse model for human diseases. In mice, there are two CD55 (or DAF) proteins (one GPI-linked and the other expressed as a transmembrane protein [132, 133]), two CD59 GPI-linked proteins (*mCd59a* [134] and *mCd59b* [135]), and Crry, a structural and functional analog of human CD46 and CD55 that restricts three pathway convertases [136]. In contrast, humans express only one *CD55* and one *CD59.* As *Crry* is expressed only in mice and rats, it is an appropriate and relevant inhibitor for studying C3 blockade in rodents. Of note, Crry is a structural and functional ortholog of human CR1, a soluble form of which has been shown to be safe in humans. mCd59a is the primary source of CD59 anti-MAC activity in mice under physiological conditions [137, 138]. mCd59b is expressed lower level on erythrocytes and a higher level on testis as compared with mCd59a [139, 138, 140]. Also, a recent study found that non–GPI-anchored intracellular isoforms of human CD59 and mouse CD59b are implicated in normal insulin secretion [141].

To our knowledge, there are no single-gene knockout studies of the complement system for SARS-CoV-2. Using pathway-specific, anaphylatoxin, MAC, and other immune system-related knockout models with the MA30 strain-infected B6 mice or hACE2-K18 model can provide valuable insight into the specific functions, components, and regulators that contribute to complement-mediated pathogenesis. For instance, to define how the complement system is activated, three complement pathways specific knockout mice including the mice deficiency in C1q [142], MBL [143], Factor B [144] and Factor D [145], key components for the classical, lectin, and alternative pathway activations can be used for conducting in vivo studies respectively. C3, C4 and C5 knockout mice together with Crry, DAF, C3aR and/or C5aR1 and C5aR2 knockout can be used for defining the role of complement in general and C3a-C3aR and C5a-C5aR signaling in the pathogenesis of severe COVID-19 and long COVID-19 [136, 146–151]. C5, C7 and C9 and mCd59 knockouts can be used to define the role of MAC in severe COVID-19 [152–156]. In addition to cell lysis, MAC formation has a sub-lethal role in activating the inflammasome [157]. A study comparing caspase-1 (a complement-independent proinflammatory enzyme) knockout mice and wild mice would illuminate which proinflammatory mechanisms are most relevant in severe COVID-19. Conditional C3 or C5 knockout mice are useful to dissect the role of local complement activation and production in the COVID-19 pathogenesis [158]. A systemic investigation of complement knockouts together with specific pathway therapeutic inhibitors as described above may unveil non-canonical functions of the complement system and improve our understanding of COVID-19, other respiratory viral diseases as we will discuss below and SARS for future pandemics.

### The role of the complement system in other respiratory viral diseases

The role of complement in the pathogenesis of other respiratory viral infections, including RSV and influenza, has been investigated. The net impact of the role of the complement seems to vary between different disease contexts; summarizing its impact across similar diseases may provide a beneficial context for the case of COVID-19.

#### The detrimental role of the complement in RSV infection

Like in COVID-19, complement hyperactivation also plays a prominent role in the context of RSV. RSV is a common viral infection that primarily affects the respiratory tract, particularly in young children. RSV is a member of the Paramyxoviridae family, with a (-)ssRNA genome. Much like COVID-19, healthy individuals respond to RSV by developing mild cold-like symptoms, while infants, older adults, and immunocompromised individuals may develop more severe respiratory illness including bronchiolitis and pneumonia.

In one mouse model simulating enhanced RSV disease, a particularly severe manifestation of RSV, researchers found symptoms were abrogated in C3-deficient mice [159] (Table 1). Prior to infection, these mice had been administered an RSV vaccine which had previously been associated with increased morbidity in human children. While histopathology revealed similar levels of lung inflammation, C3-deficient mice had much lower measured airway hyperresponsiveness compared to their wild-type counterparts [159]. High airway hyperresponsiveness persists in C5-deficient mice, highlighting the effects of C3a in particular [159]. This hypothesis was further corroborated by a separate study investigating the inhibition of the C3a-C3aR axis in RSV-infected mice [6] (Table 1). They found that C3aR1-deficient RSV-infected mice had similar levels of airway resistance to healthy (sham-infected) controls. These mice had lower inflammation and faster viral clearance than wild-type infected mice. C3aR-deficient mice transplanted with wild-type bone marrow exhibited wild-type airway hyperresponsiveness, while wild-type mouse recipients with C3aR−/− donors exhibited a rescue phenotype, highlighting the importance of bone marrow-derived immune cells in complement overactivation. Molecular profiling revealed heightened production of Th17 cytokines as a potential mechanism for acute RSV-associated pathophysiology via complement C3a and tachykinins [6].

In another study, BALB/c mice were administered cobra venom factor to deplete complement components by activating the complement system [160]. Viral titers from isolated lungs revealed similar viral expression between complement-deficient and normal mice; however, the complement system may be more adaptive in repeat infections of RSV, since the complement system appears to assist in the antibody-mediated restriction of RSV replication [160] (Table 1). This suggests that the complement system does not seem to prevent RSV replication in infection-naïve mice. Additionally, RSV can escape immune surveillance via the incorporation of complement regulators into viral particles. One in vitro study revealed that CD55 KO (but not CD46 KO) cell lines had greater affinity to C3 deposition and had 2–3 times higher percentages of opsonized viral particles compared to wild-type cells [161]. Viral camouflage via host complement regulators combined with a direct contribution to harmful pathologies like airway hyperresponsiveness reduces the efficacy of complement activity in RSV. This may serve as a parallel for COVID-19. Indeed, some evidence suggests that gC1qR may play a role both in camouflaging COVID-19 viral particles and acting as a platform for complement activation and C3a and C5a production [70].

#### The role of complement in influenza infection

While cytokine storm and complement overactivation are issues in influenza A [162, 163], evidence suggests that the complement plays a more complicated role in corresponding immune response as compared to coronavirus and RSV infections. Influenza, colloquially referred to as the flu, is caused by viruses of the Orthomyxoviridae family. Various studies have highlighted the complement system’s ability to contribute to defense against influenza and control infection. C3-deficient mice show delayed viral clearance and increased viral loads in the lung. Fewer CD4 + and CD8 + T-cells were detected in the BALF of these mice, indicating that C3 plays an important role in promoting specific immunity during influenza infection via the induction of T-cell lymphocyte priming and migration [8] (Table 1). Another study of C3 knockout mice infected with avian influenza (H5N1) produced similar results. C3 deficiency was again associated with delayed viral clearance, along with higher morbidity, as measured by decreased body weight, and greater histological damage [164] (Table 1). These experimental results indicate that C3 has a protective role in influenza infection.

In contrast, Sun et al. reported that treatment of H5N1-infected mice with a C3aR antagonist, anti-C5a antibody or with cobra venom factor significantly reduced acute lung injury [163]. Consistently, using C5aR1 deficient mice and the mice treated with an anti-C5aR1 antibody, Song et al. also show that C5a-C5aR1 signaling activation contributes to the development of influenza-induced acute lung injury [162] (Table 1). The removal of regulators like CD59 (CD59a-/-) in mice infected with influenza is associated with increased lung inflammation [165] (Table 1). However, treatment of mice with soluble complement receptor 1 reduced levels of lung-infiltrating neutrophils but not CD4 + T-cells, suggesting that canonical complement regulators may be associated with non-terminal activation and complement-independent pathways [165]. These results indicate that excessive complement activation accelerates H5N1-induced acute lung injury [163]. A better understanding of complement camouflage and the mechanistic role of complement activity in different pathologies may prove vital in managing future respiratory viral diseases.

#### Other respiratory diseases

Among other respiratory diseases, rhinoviruses, a diverse group of Picornaviridae responsible for the common cold, are known to inhibit intracellular complement activation (via the C3 signaling mechanism) through the expression of a viral cytosolic 3C protease [166, 167]. As nonenveloped viruses, intracellular complement signaling is particularly important. Interestingly, cell studies have revealed that intracellular C3 activation is basal during infection with RSV, an enveloped virus. Cell studies have also revealed that rupintrivir, a 3C protease inhibitor, can restore intracellular complement sensing and normalize complement activation [167].

## Conclusions and future directions

The complement system plays diverse roles in the pathogenesis of respiratory viral infections. It contributes to host defense via the antibody-mediated complement-dependent lysis of virions and infected cells, helps neutralize viral particles, and primes the adaptive immune system by inducing T-cell lymphocytes; however, it also exacerbates morbidity and mortality via the hyperinflammatory effects of complement components, such as the anaphylatoxins C3a and C5a and the formation of MAC. While these maladaptive effects are particularly severe in COVID-19 and RSV, the role of the complement system in influenza A is complicated (namely, C3 may be protective while C3a-C3aR, C5a-C5aR may be harmful to the host). The mechanisms responsible for this difference remain unclear. Our current understanding leaves us with three broad questions:

1. What mediates the complement system’s deleterious effects in coronaviruses and RSV compared to its impact on influenza infection?
2. What are the mechanisms by which SARS-CoV-2 and RSV viral particles evade the complement system?
3. What are the relative roles of the activation of the three complement pathways in COVID-19 and other respiratory viral diseases?

Addressing these questions requires a comparison study such as flu vs COVID-19 infection in animal models [168] together with complement knockout mouse models as discussed above and a proteomic approach. Clinical observations also fail to differentiate between sub-lytic and lethal MAC formation. While C5 inhibition via eculizumab has been successful in treating these patients, the mechanism of damage must be clarified before better-targeted therapeutics can be designed. While clinical trials have shown some success modulating severe COVID-19 with monoclonal antibodies used to treat similar complement-related diseases, the lack of a fundamental understanding of the complement system precludes the development of more efficacious treatments. The following research questions will reveal the mechanistic roles of complement components in the pathogenesis of viral respiratory diseases:

1. What are the cellular mechanisms underlying complement-accelerated COVID-19? Do complement-mediated platelet activation, thrombosis, endothelial cell dysfunction, and/or myeloid cell activation contribute to COVID-19?
2. How do the anaphylactic, opsonizing, and terminal arms of the complement contribute to COVID-19 pathogenesis?
3. How does local complement production contribute to disease pathology compared to liver-derived components?
4. How does intracellular complement activation functionally differ from activation on the membrane/in plasma? Does this vary by cell type or disease context?
5. How do C3a, C5a, and sublytic MAC formation contribute to disease?

Addressing these questions requires further experimental investigation via animal models together with other approaches such as cell ablation, single-cell and special transcriptomics, and proteomics analysis [168–171]. Complement-related loss-of-function and conditional knockout models across the complement cascade, including organ-based (lung/liver) complement deficiencies, should be extensively studied and compared against pre-existing models. A thorough investigation of complement activation and regulation may provide further targets for therapy and build a strong foundation to defend against future pandemics.

## Data Availability

Not applicable.

## References

[1] Liu F, Dai S, Gordon J, Qin X (2014). Complement and HIV-I infection/HIV-associated neurocognitive disorders. J Neurovirol.

[2] Acosta J, Qin X, Halperin J (2004). Complement and complement regulatory proteins as potential molecular targets for vascular diseases. Curr Pharm Des.

[3] Ehrnthaller C, Ignatius A, Gebhard F, Huber-Lang M (2011). New insights of an old defense system: structure, function, and clinical relevance of the complement system. Mol Med.

[4] Garred P, Tenner AJ, Mollnes TE (2021). Therapeutic targeting of the complement system: from rare diseases to pandemics. Pharmacol Rev.

[5] Heesterbeek DAC, Angelier ML, Harrison RA, Rooijakkers SHM (2018). Complement and bacterial infections: from molecular mechanisms to therapeutic applications. J Innate Immun.

[6] Bera MM, Lu B, Martin TR (2011). Th17 cytokines are critical for respiratory syncytial virus-associated airway hyperreponsiveness through regulation by complement C3a and tachykinins. J Immunol.

[7] Datta PK, Liu F, Fischer T, Rappaport J, Qin X (2020). SARS-CoV-2 pandemic and research gaps: understanding SARS-CoV-2 interaction with the ACE2 receptor and implications for therapy. Theranostics.

[8] Kopf M, Abel B, Gallimore A, Carroll M, Bachmann MF (2002). Complement component C3 promotes T-cell priming and lung migration to control acute influenza virus infection. Nat Med.

[9] Rattan A, Pawar SD, Nawadkar R (2017). Synergy between the classical and alternative pathways of complement is essential for conferring effective protection against the pandemic influenza A(H1N1) 2009 virus infection. PLoS Pathog.

[10] Nonaka M, Kimura A (2006). Genomic view of the evolution of the complement system. Immunogenetics.

[11] Sarma JV, Ward PA (2011). The complement system. Cell Tissue Res.

[12] Zhou X, Hu W, Qin X (2008). The role of complement in the mechanism of action of rituximab for B-cell lymphoma: implications for therapy. Oncologist.

[13] Morgan BP (1999). Regulation of the complement membrane attack pathway. Crit Rev Immunol.

[14] Cooper NR (1985). The classical complement pathway: activation and regulation of the first complement component. Adv Immunol.

[15] Dangl JL, Wensel TG, Morrison SL, Stryer L, Herzenberg LA, Oi VT (1988). Segmental flexibility and complement fixation of genetically engineered chimeric human, rabbit and mouse antibodies. EMBO J.

[16] Isenman DE, Dorrington KJ, Painter RH (1975). The structure and function of immunoglobulin domains. II. The importance of interchain disulfide bonds and the possible role of molecular flexibility in the interaction between immunoglobulin G and complement. J Immunol.

[17] Beltrame MH, Catarino SJ, Goeldner I, Boldt AB, de Messias-Reason IJ (2014). The lectin pathway of complement and rheumatic heart disease. Front Pediatr.

[18] Qin X, Gao B (2006). The complement system in liver diseases. Cell Mol Immunol.

[19] Morgan BP, Harris CL (1999). Complement regulatory proteins.

[20] Walport MJ (2001). Complement. First of two parts. N Engl J Med.

[21] Walport MJ (2001). Complement. Second of two parts. N Engl J Med.

[22] Carney DF, Lang TJ, Shin ML (1990). Multiple signal messengers generated by terminal complement complexes and their role in terminal complexes elimination. J Immunol.

[23] Papadimitriou JC, Ramm LE, Drachenberg CB, Trump BF, Shin ML (1991). Quantitative analysis of adenine nucleotides during the prelytic phase of cell death mediated by C5b-9. J Immunol.

[24] Niculescu F, Rus H, van Biesen T, Shin ML (1997). Activation of Ras and mitogen-activated protein kinase pathway by terminal complement complexes is G protein dependent. J Immunol.

[25] Niculescu F, Rus H (1999). Complement activation and atherosclerosis. Mol Immunol Sep-Oct.

[26] Niculescu F, Badea T, Rus H (1999). Sublytic C5b-9 induces proliferation of human aortic smooth muscle cells: role of mitogen activated protein kinase and phosphatidylinositol 3-kinase. Atherosclerosis.

[27] Niculescu F, Rus H (2004). The role of complement activation in atherosclerosis. Immunol Res.

[28] Hila S, Soane L, Koski CL (2001). Sublytic C5b–9-stimulated Schwann cell survival through PI 3-kinase-mediated phosphorylation of BAD. Glia.

[29] Soane L, Cho HJ, Niculescu F, Rus H, Shin ML (2001). C5b-9 terminal complement complex protects oligodendrocytes from death by regulating Bad through phosphatidylinositol 3-kinase/Akt pathway. J Immunol.

[30] Fosbrink M, Niculescu F, Rus V, Shin ML, Rus H (2006). C5b–9-induced endothelial cell proliferation and migration are dependent on Akt inactivation of forkhead transcription factor FOXO1. J Biol Chem.

[31] Benzaquen LR, Nicholson-Weller A, Halperin JA (1994). Terminal complement proteins C5b–9 release basic fibroblast growth factor and platelet-derived growth factor from endothelial cells. J Exp Med.

[32] Nicholson-Weller A, Halperin JA (1993). Membrane signaling by complement C5b–9, the membrane attack complex. Immunol Res.

[33] Wu G, Chen T, Shahsafaei A (2010). Complement regulator CD59 protects against angiotensin II-induced abdominal aortic aneurysms in mice. Circulation.

[34] Wu G, Hu W, Shahsafaei A (2009). Complement regulator CD59 protects against atherosclerosis by restricting the formation of complement membrane attack complex. Circ Res.

[35] Liszewski MK, Kolev M, Le Friec G (2013). Intracellular complement activation sustains T cell homeostasis and mediates effector differentiation. Immunity.

[36] Arbore G, Kemper C, Kolev M (2017). Intracellular complement—the complosome—in immune cell regulation. Mol Immunol.

[37] Reichhardt MP, Meri S (2018). Intracellular complement activation—An alarm raising mechanism?. Semin Immunol.

[38] Ferreira VP, Pangburn MK, Cortés C (2010). Complement control protein factor H: the good, the bad, and the inadequate. Mol Immunol.

[39] Risitano AM (2013). Paroxysmal nocturnal hemoglobinuria and the complement system: recent insights and novel anticomplement strategies. Adv Exp Med Biol.

[40] Joseph C, Gattineni J (2013). Complement disorders and hemolytic uremic syndrome. Curr Opin Pediatr.

[41] Lynch PJ (2006) Lungs-simple diagram of lungs and trachea. Wikipedia

[42] Lynch PJ (2021) Heart anterior exterior view. Wikipedia

[43] Cole M Image of Liver. Vecteezy.com

[44] National Center for PTSD USDoV (2021) SARS-CoV-2 particle diagram. brainline2021

[45] TefiM (2017) Healthy human elastic artery, detailed illustration

[46] Hu W, Ge X, You T (2011). Human CD59 inhibitor sensitizes rituximab-resistant lymphoma cells to complement-mediated cytolysis. Cancer Res.

[47] Zhang R, Liu Q, Liao Q, Zhao Y (2018). CD59: a promising target for tumor immunotherapy. Future Oncol.

[48] Saifuddin M, Parker CJ, Peeples ME (1995). Role of virion-associated glycosylphosphatidylinositol-linked proteins CD55 and CD59 in complement resistance of cell line-derived and primary isolates of HIV-1. J Exp Med.

[49] Nguyen DH, Hildreth JE (2000). Evidence for budding of human immunodeficiency virus type 1 selectively from glycolipid-enriched membrane lipid rafts. J Virol.

[50] Stoiber H, Pruenster M, Ammann CG, Dierich MP (2005). Complement-opsonized HIV: the free rider on its way to infection. Mol Immunol.

[51] Rautemaa R, Helander T, Meri S (2002). Herpes simplex virus 1 infected neuronal and skin cells differ in their susceptibility to complement attack. Immunology.

[52] Bernet J, Mullick J, Singh AK, Sahu A (2003). Viral mimicry of the complement system. J Biosci.

[53] Yu Q, Yu R, Qin X (2010). The good and evil of complement activation in HIV-1 infection. Cell Mol Immunol.

[54] Java A, Apicelli AJ, Liszewski MK (2020). The complement system in COVID-19: friend and foe?. JCI Insight.

[55] Que Y, Hu C, Wan K (2022). Cytokine release syndrome in COVID-19: a major mechanism of morbidity and mortality. Int Rev Immunol.

[56] Skendros P, Mitsios A, Chrysanthopoulou A (2020). Complement and tissue factor-enriched neutrophil extracellular traps are key drivers in COVID-19 immunothrombosis. J Clin Invest.

[57] Sinkovits G, Mező B, Réti M (2021). Complement overactivation and consumption predicts in-hospital mortality in SARS-CoV-2 infection. Front Immunol.

[58] Rambaldi A, Gritti G, Micò MC (2020). Endothelial injury and thrombotic microangiopathy in COVID-19: Treatment with the lectin-pathway inhibitor narsoplimab. Immunobiology.

[59] Ma L, Sahu SK, Cano M (2021). Increased complement activation is a distinctive feature of severe SARS-CoV-2 infection. Sci Immunol..

[60] Devalaraja-Narashimha K, Ehmann PJ, Huang C (2023). Association of complement pathways with COVID-19 severity and outcomes. Microb Infect.

[61] Holter JC, Pischke SE, de Boer E (2020). Systemic complement activation is associated with respiratory failure in COVID-19 hospitalized patients. Proc Natl Acad Sci.

[62] Giavedoni P, Podlipnik S, Pericàs JM (2020). Skin manifestations in COVID-19: prevalence and relationship with disease severity. J Clin Med.

[63] Carvelli J, Demaria O, Vély F (2020). Association of COVID-19 inflammation with activation of the C5a–C5aR1 axis. Nature.

[64] Macor P, Durigutto P, Mangogna A (2021). Multiple-organ complement deposition on vascular endothelium in COVID-19 patients. Biomedicines.

[65] Robbins RA, Russ WD, Rasmussen JK, Clayton MM (1987). Activation of the complement system in the adult respiratory distress syndrome. Am Rev Respir Dis.

[66] Zilow G, Joka T, Obertacke U, Rother U, Kirschfink M (1992). Generation of anaphylatoxin C3a in plasma and bronchoalveolar lavage fluid in trauma patients at risk for the adult respiratory distress syndrome. Crit Care Med.

[67] Hammerschmidt DE, Weaver LJ, Hudson LD, Craddock PR, Jacob HS (1980). Association of complement activation and elevated plasma-C5a with adult respiratory distress syndrome. Pathophysiological relevance and possible prognostic value. Lancet.

[68] Peffault de Latour R, Bergeron A, Lengline E (2020). Complement C5 inhibition in patients with COVID-19—a promising target?. Haematologica.

[69] Rajamanickam A, Nathella PK, Venkataraman A (2023). Levels of complement components in children with acute COVID-19 or multisystem inflammatory syndrome. JAMA Netw Open.

[70] Savitt AG, Manimala S, White T (2021). SARS-CoV-2 exacerbates COVID-19 pathology through activation of the complement and kinin systems. Front Immunol.

[71] Afzali B, Noris M, Lambrecht BN, Kemper C (2022). The state of complement in COVID-19. Nat Revi Immunol.

[72] Legrand M, Bell S, Forni L (2021). Pathophysiology of COVID-19-associated acute kidney injury. Nat Rev Nephrol.

[73] Pfister F, Vonbrunn E, Ries T (2020). Complement activation in kidneys of patients with COVID-19. Front Immunol.

[74] Santana MF, Guerra MT, Hundt MA (2022). Correlation between clinical and pathological findings of liver injury in 27 patients with lethal COVID-19 infections in Brazil. Hepatol Commun.

[75] Pellegrini D, Kawakami R, Guagliumi G (2021). Microthrombi as a major cause of cardiac injury in COVID-19. Circulation.

[76] Magro CM, Mulvey JJ, Laurence J (2021). The differing pathophysiologies that underlie COVID-19-associated perniosis and thrombotic retiform purpura: a case series. Br J Dermatol.

[77] Ville S, Le Bot S, Chapelet-Debout A (2021). Atypical HUS relapse triggered by COVID-19. Kidney Int.

[78] Kaufeld J, Reinhardt M, Schröder C (2021). Atypical hemolytic and uremic syndrome triggered by infection with SARS-CoV2. Kidney Int Rep.

[79] Otieno SB, Altahan A, Kaweeta F, Karri S, Alnoor F, Johnson R (2021). Severe hemolysis in a COVID-19 patient with paroxysmal nocturnal hemoglobinuria. Case Rep Hematol.

[80] Hines A, Hakim N, Barrientos J (2021). COVID-19 infection presenting as paroxysmal nocturnal hemoglobinuria. Clin Case Rep.

[81] Uwatoko R, Shindo M, Hashimoto N (2023). Relapse of atypical hemolytic uremic syndrome triggered by COVID-19: a lesson for the clinical nephrologist. J Nephrol.

[82] Sinkovits G, Schnur J, Hurler L (2022). Evidence, detailed characterization and clinical context of complement activation in acute multisystem inflammatory syndrome in children. Sci Rep.

[83] Ziegler CGK, Allon SJ, Nyquist SK (2020). SARS-CoV-2 receptor ACE2 is an interferon-stimulated gene in human airway epithelial cells and is detected in specific cell subsets across tissues. Cell.

[84] Zhang H, Gerasimovskaya E, McCarthy MK (2023). Local complement contributes to pathogenic activation of lung endothelial cells in SARS-CoV-2 infection. Am J Respir Cell Mol Biol.

[85] Navratil JS, Watkins SC, Wisnieski JJ, Ahearn JM (2001). The globular heads of C1q specifically recognize surface blebs of apoptotic vascular endothelial cells. J Immunol.

[86] Albertí S, Marqués G, Camprubí S (1993). C1q binding and activation of the complement classical pathway by Klebsiella pneumoniae outer membrane proteins. Infect Immun.

[87] Beirag N, Varghese PM, Neto MM (2023). Complement activation-independent attenuation of SARS-CoV-2 infection by C1q and C4b-binding protein. Viruses.

[88] Varghese PM, Kishore U, Rajkumari R (2022). Human C1q regulates influenza A virus infection and inflammatory response via its globular domain. Int J Mol Sci.

[89] Hurler L, Szilágyi Á, Mescia F (2023). Complement lectin pathway activation is associated with COVID-19 disease severity, independent of MBL2 genotype subgroups Original Research. Front Immunol.

[90] Gao T, Zhu L, Liu H (2022). Highly pathogenic coronavirus N protein aggravates inflammation by MASP-2-mediated lectin complement pathway overactivation. Signal Transduct Targeted Therapy.

[91] Yu J, Yuan X, Chen H, Chaturvedi S, Braunstein EM, Brodsky RA (2020). Direct activation of the alternative complement pathway by SARS-CoV-2 spike proteins is blocked by factor D inhibition. Blood.

[92] Ali YM, Carnell GW, Fumagalli S (2023). Inhibition of the lectin pathway of complement activation reduces acute respiratory distress syndrome severity in a mouse model of SARS-CoV-2 infection. J Infect Dis.

[93] Queiroz MAF, Santiago AM, Brito W (2023). Polymorphisms in the MBL2 gene are associated with the plasma levels of MBL and the cytokines IL-6 and TNF-α in severe COVID-19. Front Immunol.

[94] Waters AM, Licht C (2011). aHUS caused by complement dysregulation: new therapies on the horizon. Pediatr Nephrol.

[95] Siggins MK, Davies K, Fellows R (2023). Alternative pathway dysregulation in tissues drives sustained complement activation and predicts outcome across the disease course in COVID-19. Immunology.

[96] Tsiftsoglou SA, Gavriilaki E, Touloumenidou T (2023). Targeted genotyping of COVID-19 patients reveals a signature of complement C3 and factor B coding SNPs associated with severe infection. Immunobiology.

[97] Kawakami E, Saiki N, Yoneyama Y (2023). Complement factor D targeting protects endotheliopathy in organoid and monkey models of COVID-19. Cell Stem Cell.

[98] Kumar J, Dhyani S, Kumar P, Sharma NR, Ganguly S (2023). SARS-CoV-2-encoded ORF8 protein possesses complement inhibitory properties. J Biol Chem.

[99] Sahu SK, Ozantürk AN, Kulkarni DH (2023). Lung epithelial cell-derived C3 protects against pneumonia-induced lung injury. Sci Immunol.

[100] Wang S, Yao X, Ma S (2021). A single-cell transcriptomic landscape of the lungs of patients with COVID-19. Nat Cell Biol.

[101] Chouaki Benmansour N, Carvelli J, Vivier E (2021). Complement cascade in severe forms of COVID-19: recent advances in therapy. Eur J Immunol.

[102] Ruggenenti P, Di Marco F, Cortinovis M (2021). Eculizumab in patients with severe coronavirus disease 2019 (COVID-19) requiring continuous positive airway pressure ventilator support: Retrospective cohort study. PLoS ONE.

[103] Annane D, Heming N, Grimaldi-Bensouda L (2020). Eculizumab as an emergency treatment for adult patients with severe COVID-19 in the intensive care unit: a proof-of-concept study. eClinicalMedicine.

[104] Diurno F (2020). Eculizumab treatment in patients with COVID-19: preliminary results from real life ASL Napoli 2 Nord experience. Eur Rev Med Pharmacol Sci.

[105] Lim EHT, van Amstel RBE, de Boer VV (2023). Complement activation in COVID-19 and targeted therapeutic options: a scoping review. Blood Rev.

[106] Zelek WM, Cole J, Ponsford MJ (2020). Complement Inhibition with the C5 Blocker LFG316 in Severe COVID-19. Am J Respir Crit Care Med.

[107] McEneny-King AC, Monteleone JPR, Kazani SD, Ortiz SR (2021). Pharmacokinetic and pharmacodynamic evaluation of ravulizumab in adults with severe coronavirus disease 2019. Infect Dis Ther.

[108] Witzenrath M, Paassen P, Heunks L (2022). Anti-C5a antibody (vilobelimab) therapy for critically ill, invasively mechanically ventilated patients with COVID-19 (PANAMO): a multicentre, double-blind, randomised, placebo-controlled, phase 3 trial. The Lancet Respir Med.

[109] Skendros P, Germanidis G, Mastellos DC (2022). Complement C3 inhibition in severe COVID-19 using compstatin AMY-101. Sci Adv.

[110] Mastellos DC, Pires da Silva BGP, Fonseca BAL (2020). Complement C3 vs C5 inhibition in severe COVID-19: early clinical findings reveal differential biological efficacy. Clin Immunol.

[111] Urwyler P, Moser S, Charitos P (2020). Treatment of COVID-19 with conestat alfa, a regulator of the complement, contact activation and Kallikrein–Kinin system. Front Immunol.

[112] Mansour E, Palma AC, Ulaf RG (2021). Safety and outcomes associated with the pharmacological inhibition of the Kinin–Kallikrein system in severe COVID-19. Viruses.

[113] Zhou Y, Lu K, Pfefferle S (2010). A single asparagine-linked glycosylation site of the severe acute respiratory syndrome coronavirus spike glycoprotein facilitates inhibition by mannose-binding lectin through multiple mechanisms. J Virol.

[114] Hamed ME, Naeem A, Alkadi H (2021). Elevated expression levels of lung complement anaphylatoxin, neutrophil chemoattractant chemokine IL-8, and RANTES in MERS-CoV-infected patients: predictive biomarkers for disease severity and mortality. J Clin Immunol.

[115] Alsolamy S, Arabi YM (2015). Infection with Middle East respiratory syndrome coronavirus. Can J Respir Ther Fall.

[116] Pormohammad A, Ghorbani S, Khatami A (2020). Comparison of confirmed COVID-19 with SARS and MERS cases—Clinical characteristics, laboratory findings, radiographic signs and outcomes: a systematic review and meta-analysis. Rev Med Virol.

[117] Raj VS, Mou H, Smits SL (2013). Dipeptidyl peptidase 4 is a functional receptor for the emerging human coronavirus-EMC. Nature.

[118] Saad M, Omrani AS, Baig K (2014). Clinical aspects and outcomes of 70 patients with Middle East respiratory syndrome coronavirus infection: a single-center experience in Saudi Arabia. Int J Infect Dis.

[119] Gralinski LE, Sheahan TP, Morrison TE (2018). Complement activation contributes to severe acute respiratory syndrome coronavirus pathogenesis. MBio.

[120] Jiang Y, Zhao G, Song N (2018). Blockade of the C5a–C5aR axis alleviates lung damage in hDPP4-transgenic mice infected with MERS-CoV. Emerg Microbes Infect.

[121] Becker K, Beythien G, de Buhr N (2021). Vasculitis and neutrophil extracellular traps in lungs of golden syrian hamsters with SARS-CoV-2. Front Immunol.

[122] Aid M, Vidal SJ, Piedra-Mora C (2022). Ad26.COV2.S prevents upregulation of SARS-CoV-2 induced pathways of inflammation and thrombosis in hamsters and rhesus macaques. PLoS Pathog.

[123] Muñoz-Fontela C, Dowling WE, Funnell SGP (2020). Animal models for COVID-19. Nature.

[124] Zheng J, Wong LR, Li K (2021). COVID-19 treatments and pathogenesis including anosmia in K18-hACE2 mice. Nature.

[125] Oladunni FS, Park JG, Pino PA (2020). Lethality of SARS-CoV-2 infection in K18 human angiotensin-converting enzyme 2 transgenic mice. Nat Commun.

[126] Qin Z, Liu F, Blair R (2021). Endothelial cell infection and dysfunction, immune activation in severe COVID-19. Theranostics.

[127] Khatun MS, Remcho TP, Qin X, Kolls JK (2023). Cell-intrinsic and -extrinsic effects of SARS-CoV-2 RNA on pathogenesis: single-cell meta-analysis. mSphere.

[128] Dong W, Mead H, Tian L (2022). The K18-human ACE2 transgenic mouse model recapitulates non-severe and severe COVID-19 in response to an infectious dose of the SARS-CoV-2 Virus. J Virol.

[129] Dong W, Mead H, Tian L (2022). The K18-Human ACE2 transgenic mouse model recapitulates non-severe and severe COVID-19 in response to an infectious dose of the SARS-CoV-2 virus. J Virol.

[130] Leist SR, Dinnon KH, Schäfer A (2020). A mouse-adapted SARS-CoV-2 induces acute lung injury and mortality in standard laboratory mice. Cell.

[131] Wong L-YR, Zheng J, Wilhelmsen K (2022). Eicosanoid signalling blockade protects middle-aged mice from severe COVID-19. Nature.

[132] Spicer AP, Seldin MF, Gendler SJ (1995). Molecular cloning and chromosomal localization of the mouse decay- accelerating factor genes Duplicated genes encode glycosylphosphatidylinositol-anchored and transmembrane forms. J Immunol.

[133] Lin F, Fukuoka Y, Spicer A (2001). Tissue distribution of products of the mouse decay-accelerating factor (DAF) genes Exploitation of a Daf1 knock-out mouse and site-specific monoclonal antibodies. Immunology.

[134] Powell MB, Marchbank KJ, Rushmere NK, van den Berg CW, Morgan BP (1997). Molecular cloning, chromosomal localization, expression, and functional characterization of the mouse analogue of human CD59. J Immunol.

[135] Qian YM, Qin X, Miwa T, Sun X, Halperin JA, Song WC (2000). Identification and functional characterization of a new gene encoding the mouse terminal complement inhibitor CD59. J Immunol.

[136] Paul MS, Aegerter M, Cepek K, Miller MD, Weis JH (1990). The murine complement receptor gene family. III. The genomic and transcriptional complexity of the Crry and Crry-ps genes. J Immunol.

[137] Baalasubramanian S, Harris CL, Donev RM (2004). CD59a is the primary regulator of membrane attack complex assembly in the mouse. J Immunol.

[138] Qin X, Dobarro M, Bedford SJ (2005). Further characterization of reproductive abnormalities in mCd59b knockout mice: a potential new function of mCd59 in male reproduction. J Immunol.

[139] Qin X, Miwa T, Aktas H (2001). Genomic structure, functional comparison, and tissue distribution of mouse Cd59a and Cd59b. Mamm Genome.

[140] Qin X, Ferris S, Hu W, Guo F, Ziegeler G, Halperin JA (2006). Analysis of the promoters and 5'-UTR of mouse Cd59 genes, and of their functional activity in erythrocytes. Genes Immun.

[141] Golec E, Ekström A, Noga M (2022). Alternative splicing encodes functional intracellular CD59 isoforms that mediate insulin secretion and are down-regulated in diabetic islets. Proc Natl Acad Sci U S A.

[142] Fonseca MI, Chu SH, Hernandez MX (2017). Cell-specific deletion of C1qa identifies microglia as the dominant source of C1q in mouse brain. J Neuroinflamm.

[143] Shi L, Takahashi K, Dundee J (2004). Mannose-binding lectin-deficient mice are susceptible to infection with *Staphylococcus aureus*. J Exp Med.

[144] Matsumoto M, Fukuda W, Circolo A (1997). Abrogation of the alternative complement pathway by targeted deletion of murine factor B. Proc Natl Acad Sci U S A.

[145] Xu Y, Ma M, Ippolito GC, Schroeder HW, Carroll MC, Volanakis JE (2001). Complement activation in factor D-deficient mice. Proc Natl Acad Sci U S A.

[146] Wessels MR, Butko P, Ma M, Warren HB, Lage AL, Carroll MC (1995). Studies of group B streptococcal infection in mice deficient in complement component C3 or C4 demonstrate an essential role for complement in both innate and acquired immunity. Proc Natl Acad Sci U S A.

[147] Wheat WH, Wetsel R, Falus A, Tack BF, Strunk RC (1987). The fifth component of complement (C5) in the mouse. Analysis of the molecular basis for deficiency. J Exp Med.

[148] Lin F, Kaminski HJ, Conti-Fine BM, Wang W, Richmonds C, Medof ME (2002). Markedly enhanced susceptibility to experimental autoimmune myasthenia gravis in the absence of decay-accelerating factor protection. J Clin Invest.

[149] Humbles AA, Lu B, Nilsson CA (2000). A role for the C3a anaphylatoxin receptor in the effector phase of asthma. Nature.

[150] Höpken UE, Lu B, Gerard NP, Gerard C (1996). The C5a chemoattractant receptor mediates mucosal defence to infection. Nature.

[151] Gerard NP, Lu B, Liu P (2005). An anti-inflammatory function for the complement anaphylatoxin C5a-binding protein, C5L2. J Biol Chem.

[152] Fu X, Ju J, Lin Z (2016). Target deletion of complement component 9 attenuates antibody-mediated hemolysis and lipopolysaccharide (LPS)-induced acute shock in mice. Sci Rep.

[153] Qin X, Hu W, Song W (2009). Generation and phenotyping of mCd59a and mCd59b double-knockout mice. Am J Hematol.

[154] Welsh KJ, Lewis CT, Boyd S, Braun MC, Actor JK (2012). Complement factor C7 contributes to lung immunopathology caused by *Mycobacterium tuberculosis*. Clin Dev Immunol.

[155] Qin X, Hu W, Song W (2009). Balancing role of nitric oxide in complement-mediated activation of platelets from mCd59a and mCd59b double-knockout mice. Am J Hematol.

[156] Holt DS, Botto M, Bygrave AE, Hanna SM, Walport MJ (2001). Morgan BP (2001) Targeted deletion of the CD59 gene causes spontaneous intravascular hemolysis and hemoglobinuria. Blood.

[157] Triantafilou K, Hughes TR, Triantafilou M, Morgan BP (2013). The complement membrane attack complex triggers intracellular Ca2+ fluxes leading to NLRP3 inflammasome activation. J Cell Sci.

[158] Kolev M, West EE, Kunz N (2020). Diapedesis-induced integrin signaling via LFA-1 facilitates tissue immunity by inducing intrinsic complement C3 expression in immune cells. Immunity.

[159] Polack FP, Teng MN, Collins PL (2002). A role for immune complexes in enhanced respiratory syncytial virus disease. J Exp Med.

[160] Bukreyev A, Yang L, Collins PL (2012). The secreted G protein of human respiratory syncytial virus antagonizes antibody-mediated restriction of replication involving macrophages and complement. J Virol.

[161] Kuppan JP, Mitrovich MD, Vahey MD (2021). A morphological transformation in respiratory syncytial virus leads to enhanced complement deposition. Elife.

[162] Song N, Li P, Jiang Y (2018). C5a receptor1 inhibition alleviates influenza virus-induced acute lung injury. Int Immunopharmacol.

[163] Sun S, Zhao G, Liu C (2013). Inhibition of complement activation alleviates acute lung injury induced by highly pathogenic avian influenza H5N1 virus infection. Am J Respir Cell Mol Biol.

[164] O'Brien KB, Morrison TE, Dundore DY, Heise MT, Schultz-Cherry S (2011). A protective role for complement C3 protein during pandemic 2009 H1N1 and H5N1 influenza A virus infection. PLoS ONE.

[165] Longhi MP, Williams A, Wise M, Morgan BP, Gallimore A (2007). CD59a deficiency exacerbates influenza-induced lung inflammation through complement-dependent and -independent mechanisms. Eur J Immunol.

[166] Tam JCH, Bidgood SR, McEwan WA, James LC (2014). Intracellular sensing of complement C3 activates cell autonomous immunity. Science.

[167] Mellors J, Tipton T, Longet S, Carroll M (2020). Viral evasion of the complement system and its importance for vaccines and therapeutics. Front Immunol.

[168] Wang C, Khatun MS, Zhang Z (2023). COVID-19 and influenza infections mediate distinct pulmonary cellular and transcriptomic changes. Commun Biol.

[169] Hu W, Ferris SP, Tweten RK (2008). Rapid conditional targeted ablation of cells expressing human CD59 in transgenic mice by intermedilysin. Nat Med.

[170] Feng D, Dai S, Liu F (2016). Cre-inducible human CD59 mediates rapid cell ablation after intermedilysin administration. J Clin Invest.

[171] Han K, Blair RV, Iwanaga N (2021). Lung expression of human angiotensin-converting enzyme 2 sensitizes the mouse to SARS-CoV-2 infection. Am J Respir Cell Mol Biol.

